# Supplementary material for: Mir204 and Mir211 suppress synovial inflammation and proliferation in rheumatoid arthritis by targeting Ssrp1
Source: eLife. 2022 Dec 13;11:e78085. doi: 10.7554/eLife.78085 (PMC9747153; doi:10.7554/eLife.78085)
Supplement: Figure 4—source data 2. [file elife-78085-fig4-data2.pptx]

## Slide 1
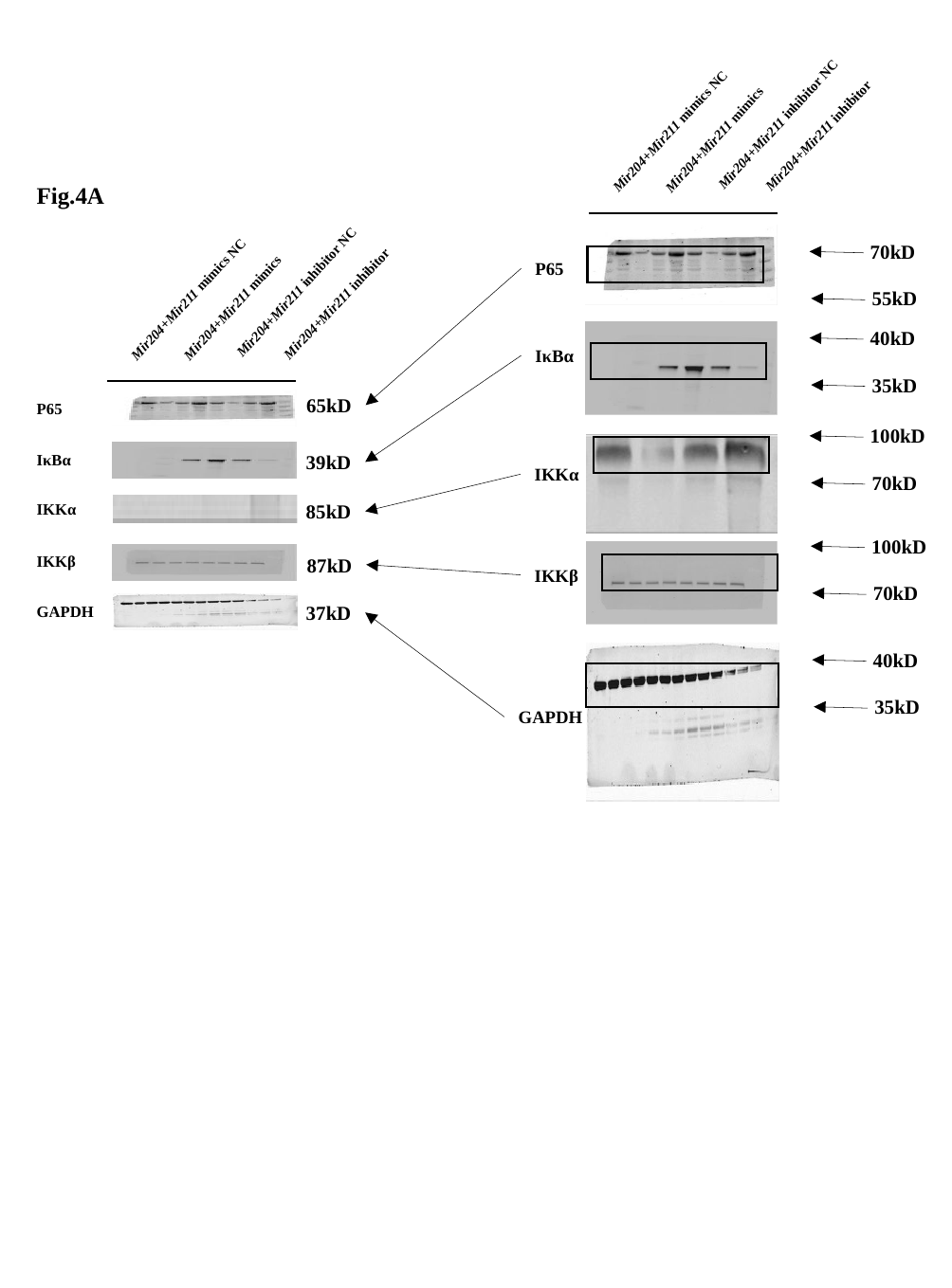

Mir204+Mir211 inhibitor NC
Mir204+Mir211 mimics NC
Mir204+Mir211 inhibitor
Mir204+Mir211 mimics
Fig.4A
70kD
P65
Mir204+Mir211 inhibitor NC
55kD
Mir204+Mir211 mimics NC
Mir204+Mir211 inhibitor
Mir204+Mir211 mimics
40kD
IκBα
35kD
65kD
P65
IκBα
IKKα
IKKβ
100kD
39kD
IKKα
70kD
85kD
100kD
87kD
IKKβ
70kD
37kD
GAPDH
40kD
35kD
GAPDH
