# Supplementary material for: Mir204 and Mir211 suppress synovial inflammation and proliferation in rheumatoid arthritis by targeting Ssrp1
Source: eLife. 2022 Dec 13;11:e78085. doi: 10.7554/eLife.78085 (PMC9747153; doi:10.7554/eLife.78085)
Supplement: Figure 5—source data 2. [file elife-78085-fig5-data2.pptx]

## Slide 1
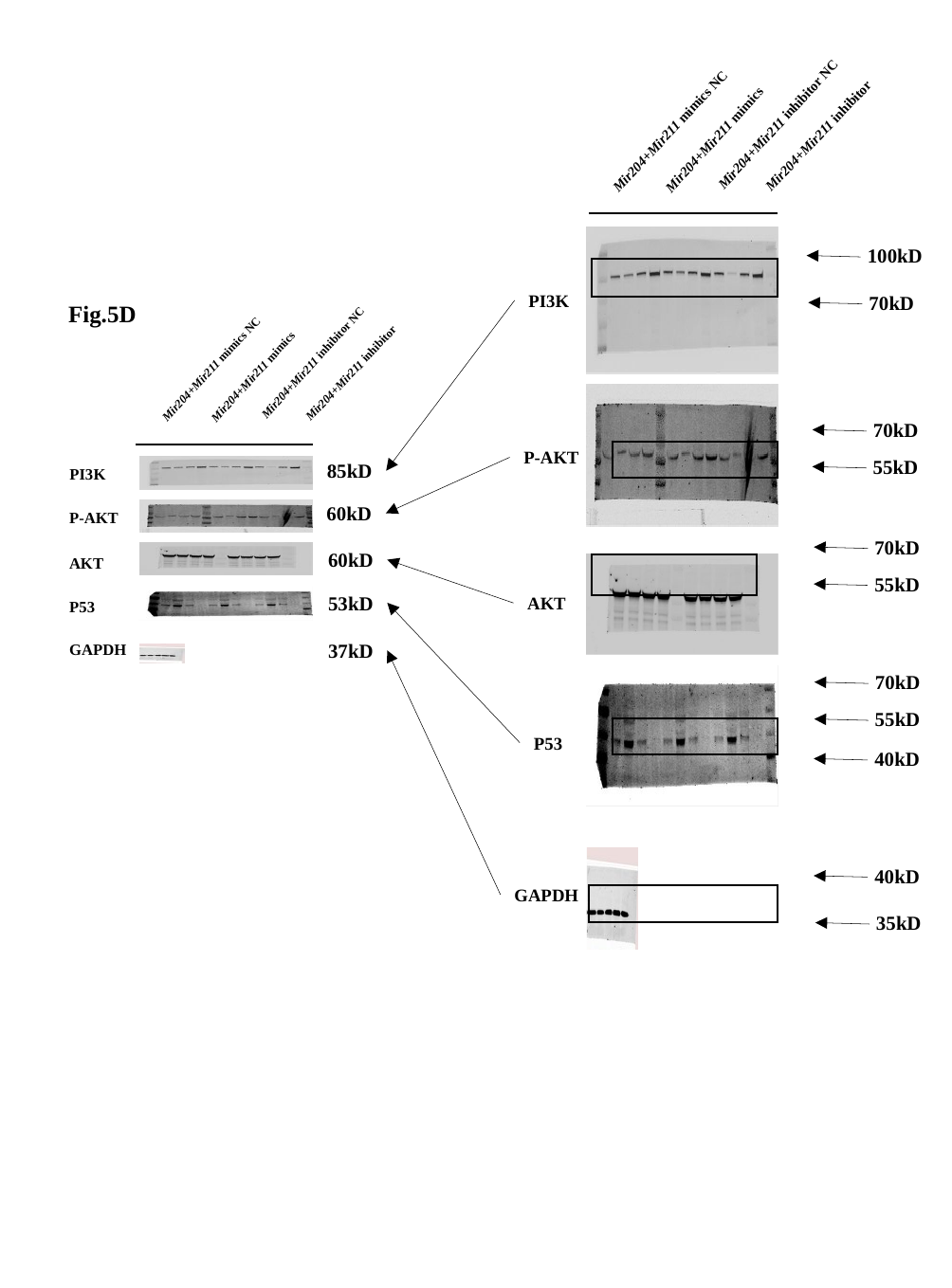

Mir204+Mir211 inhibitor NC
Mir204+Mir211 mimics NC
Mir204+Mir211 inhibitor
Mir204+Mir211 mimics
100kD
Mir204+Mir211 inhibitor NC
Mir204+Mir211 mimics NC
Mir204+Mir211 inhibitor
Mir204+Mir211 mimics
PI3K
P53
P-AKT
AKT
GAPDH
PI3K
70kD
Fig.5D
70kD
P-AKT
55kD
85kD
60kD
70kD
60kD
55kD
53kD
AKT
37kD
70kD
55kD
P53
40kD
40kD
GAPDH
35kD
