# Supplementary material for: Mir204 and Mir211 suppress synovial inflammation and proliferation in rheumatoid arthritis by targeting Ssrp1
Source: eLife. 2022 Dec 13;11:e78085. doi: 10.7554/eLife.78085 (PMC9747153; doi:10.7554/eLife.78085)
Supplement: Figure 6—source data 2. [file elife-78085-fig6-data2.pptx]

## Slide 1
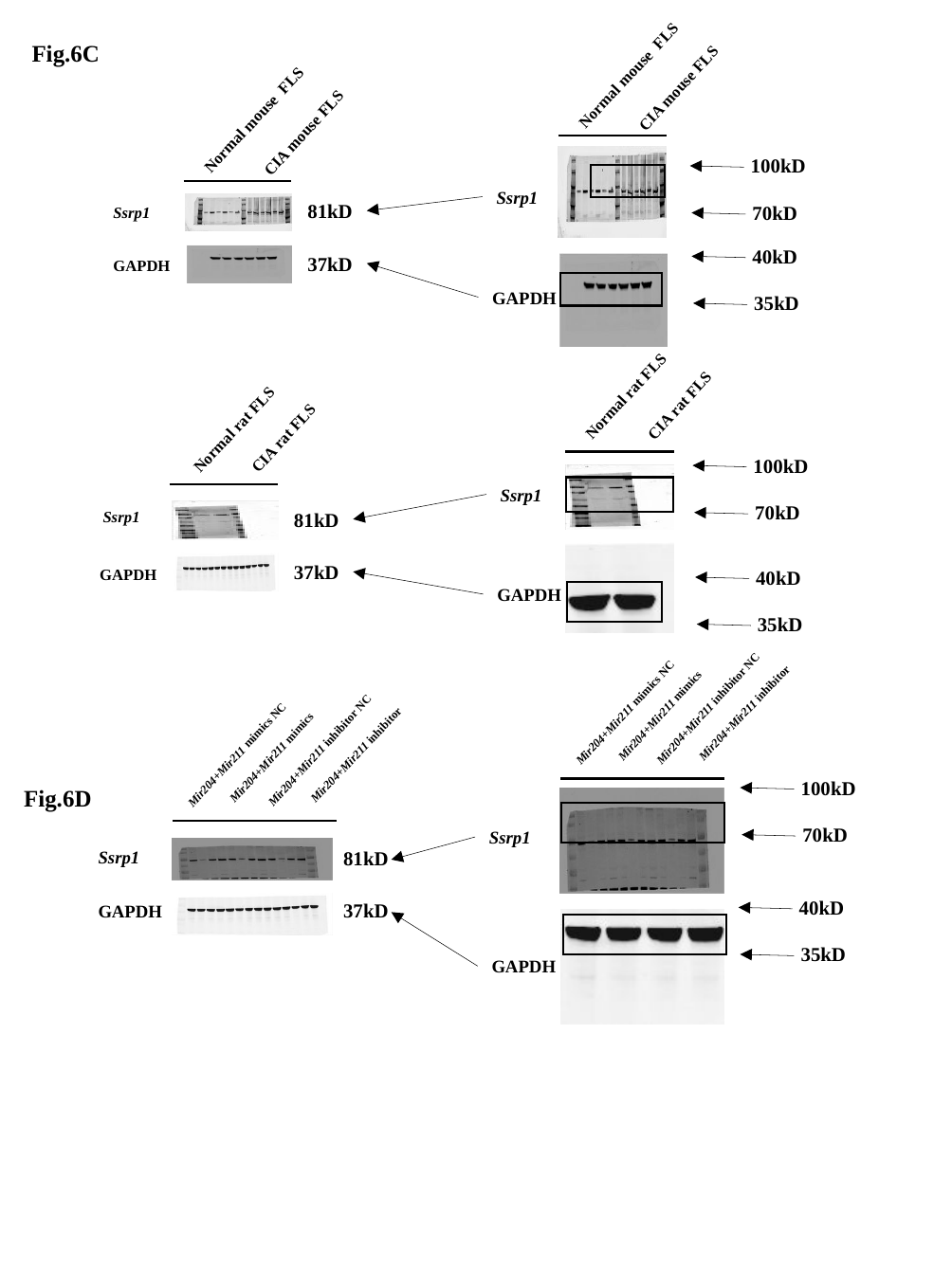

Normal mouse FLS
CIA mouse FLS
Ssrp1
GAPDH
Fig.6C
Normal mouse FLS
CIA mouse FLS
100kD
Ssrp1
81kD
70kD
40kD
37kD
GAPDH
35kD
Normal rat FLS
CIA rat FLS
Ssrp1
GAPDH
Normal rat FLS
CIA rat FLS
100kD
Ssrp1
70kD
81kD
37kD
40kD
GAPDH
35kD
Mir204+Mir211 inhibitor NC
Mir204+Mir211 inhibitor
Mir204+Mir211 mimics NC
Mir204+Mir211 mimics
Mir204+Mir211 inhibitor NC
Mir204+Mir211 inhibitor
Mir204+Mir211 mimics NC
Mir204+Mir211 mimics
100kD
Fig.6D
70kD
Ssrp1
Ssrp1
GAPDH
81kD
40kD
37kD
35kD
GAPDH
