# Supplementary material for: Mir204 and Mir211 suppress synovial inflammation and proliferation in rheumatoid arthritis by targeting Ssrp1
Source: eLife. 2022 Dec 13;11:e78085. doi: 10.7554/eLife.78085 (PMC9747153; doi:10.7554/eLife.78085)
Supplement: Figure 7—source data 2. [file elife-78085-fig7-data2.pptx]

## Slide 1
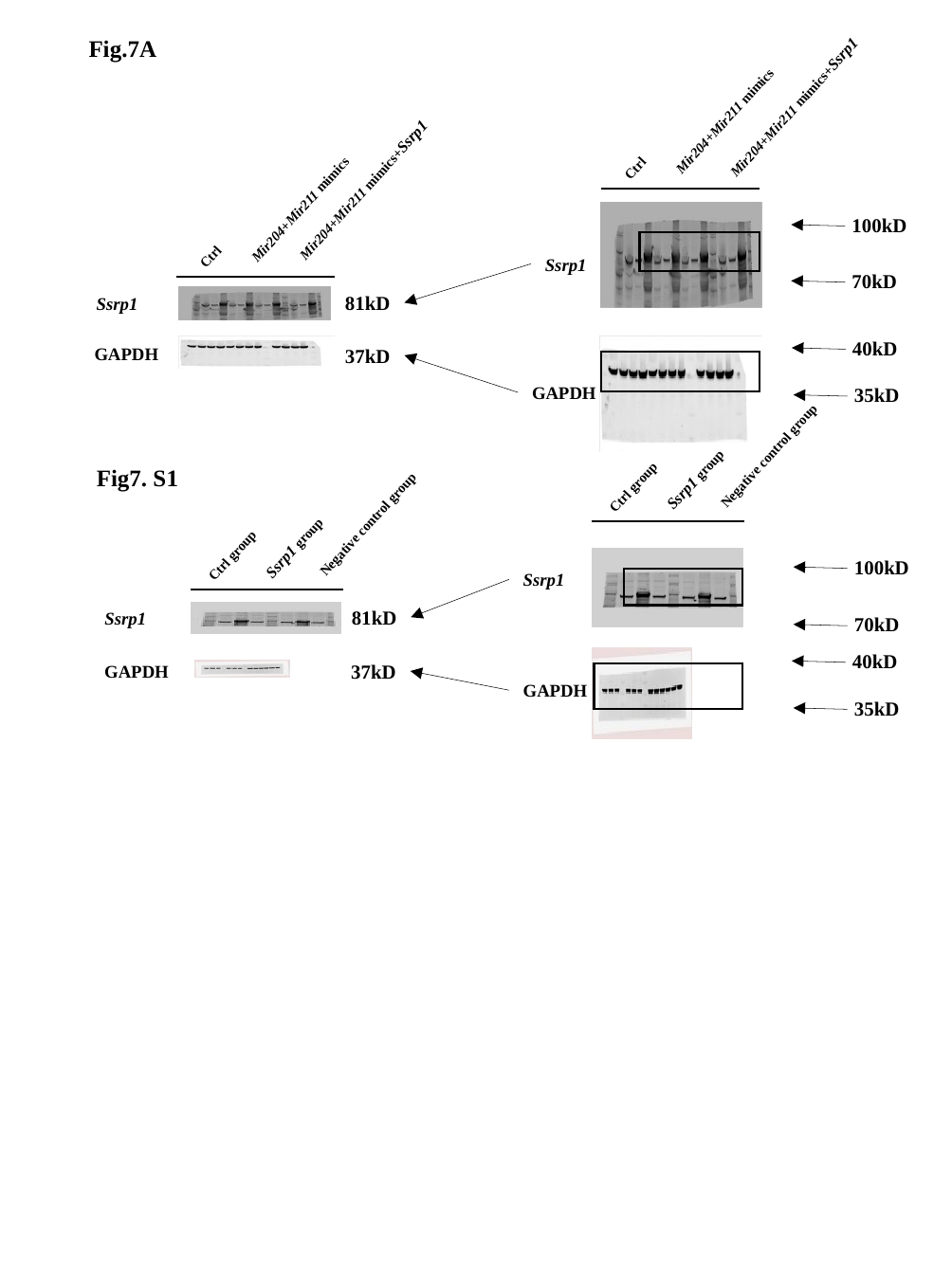

Fig.7A
Mir204+Mir211 mimics+Ssrp1
Mir204+Mir211 mimics
Ctrl
Ssrp1
GAPDH
Mir204+Mir211 mimics+Ssrp1
Mir204+Mir211 mimics
Ctrl
100kD
Ssrp1
70kD
81kD
40kD
37kD
GAPDH
35kD
Negative control group
Negative control group
Ssrp1 group
Ctrl group
Ssrp1
GAPDH
Fig7. S1
Ssrp1 group
Ctrl group
100kD
Ssrp1
81kD
70kD
40kD
37kD
GAPDH
35kD
